# Association Between Perforating Scleral Vessel and Myopic Maculopathy: A Cross-Sectional Study of a Chinese Cohort

**DOI:** 10.3389/fmed.2021.727680

**Published:** 2022-01-06

**Authors:** Huimin Yu, Jinfu Sun, Huan Luo, Zhitao Wang, Xufang Sun

**Affiliations:** Department of Ophthalmology, Tongji Hospital, Tongji Medical College, Huazhong University of Science and Technology, Wuhan, China

**Keywords:** myopic maculopathy, perforating scleral vessels, optical coherence tomography, choroidal neovascularization, multivariate analysis

## Abstract

**Purpose:** To investigate the association between perforating scleral vessel (PSV) and different types of myopic maculopathy (MM) in a highly myopic population.

**Methods:** In total, 188 highly myopic eyes (117 participants) were enrolled. Each participant underwent detailed history taking and ocular examinations. Based on fundus photographs and optical coherence tomography, patients were subdivided into the non-MM group and MM group. Based on a new classification system (ATN), MM cases were classified as myopic atrophy maculopathy (MAM), myopic tractional maculopathy (MTM), and myopic neovascular maculopathy (MNM). The number of PSV and the macular choroidal thickness (mChT) were measured.

**Results:** Compared with non-MM group, MM group was characterized by relatively larger age (48.40 vs. 32.34; *p* < 0.001), longer axial length (AL, 29.72 vs. 27.75, *p* < 0.001), thinner mChT (52.90 vs. 122.52; *p* < 0.001), and lower PSV counts (6.73 vs. 9.47, *p* ≤ 0.001). The non-MM group had higher PSV counts in total area (0–9 mm, 9.47 vs. 6.73, *p* < 0.001) and perifovea area (3–9 mm, 7.25 vs. 4.71, *p* < 0.001) compared to the MM group. Univariate and multivariate analyses showed that PSV count had no association with MAM (*p* = 0.2419) and MTM (*p* = 0.5678). Total PSV count [odds ratio (OR) 0.78, 95% CI 0.64–0.95, *p* = 0.0149] and perifovea PSV count (OR 0.80, 95% CI 0.65–0.98, *p* = 0.0299) were both protective factors for MNM. The stratified analysis revealed that in groups with AL <28 mm, or mChT <50 μm, or mChT ≥100 μm, or eyes with cilioretinal artery, PSV count had no significant association with MNM.

**Conclusion:** Higher PSV counts in perifovea area (3–9 mm centered fovea) and total area (0–9 mm centered fovea) were protective factors for MNM, whereas PSV count had no association with MAM and MTM. These findings may provide novel insights into the mechanisms of pathologic myopia.

## Introduction

Myopia causes a huge societal burden. Pathological myopia (PM) is especially prevalent in East Asian countries (1, 2). Myopic maculopathy (MM) is the main cause of vision loss in patients with PM (3, 4). In recent years, insights into the pathogenesis of high myopia due to scleral and choroidal levels have emerged. Recent studies show that perforating scleral vessel (PSV) may be anatomically associated with lacquer crack (Lc) (5), myopic choroidal neovascularization (mCNV) (6–9), and patchy atrophy (10). Previous researchers described these phenomena and calculated the probability of PSV occurrence under specific myopic complications. However, the association between PSV and MM has not been analyzed.

Choroidal and scleral thinning are typical characteristics of highly myopic eyes. Indocyanine green angiography showed that PSV was originated from the short posterior ciliary artery (SPCA), which was important to choroidal blood supply (7). Thus, PSV count may be significantly associated to myopic choroidal change and may influence PM progression. A new classification system for MM (ATN) that integrates atrophy (A), traction (T), and neovascular (N) has been recently proposed (11). Based on the ATN classification, we explored the relationship between PSV and MM in detail. Here, we analyzed the characteristics of a highly myopic adult cohort to determine if PSV count is a risk factor for the 3 MM types.

## Methods

### Study Design

We observed a series of patients presented at Tongji Hospital, Tongji Medical College, Huazhong University of Science and Technology, from August 2020 to March 2021 with high myopia in at least 1 eye. Ethical approval for the study was granted by the medical ethics committee of Tongji Hospital, Tongji Medical College, Huazhong University of Science and Technology. The study adhered to the Declaration of Helsinki guidelines. All participants gave written informed consent. The study was registered at http://www.chictr.org.cn (registration number ChiCTR2100043611).

### History Taking and Ocular Examinations

All patients underwent a thorough history review and ocular examinations. Treatment history was recorded and classified based on the inclusion and exclusion criteria. Ocular examinations involved assessment of intraocular pressure (IOP) (NT-510, NIDEK CO., LTD., Japan), slit-lamp biomicroscopy (BP900, Haag-Streit International, Swiss), indirect ophthalmoscopy (YZ6H, 66Vision.Tech, China), axial length (AL) (AL-scan, NIDEK CO., LTD, Japan), color fundus photograph (AFC-210, Nidek Co., LTD., Japan), and spectral-domain optical coherence tomography (SD-OCT) (Spectralis OCT, Heidelberg Engineering, Germany). OCT-angiography (Spectralis OCT, Heidelberg Engineering, Germany and SVision Imaging, Henan, China), fluorescence, and indocyanine green angiography (Spectralis OCT, Heidelberg Engineering, Germany) were used to diagnose MNM where necessary.

### Inclusion and Exclusion Criteria

Participants with an AL ≥26 mm were included in the study. Exclusion criteria were (1) an IOP >21 mmHg, (2) coexisting or history of severe ocular diseases, such as dense cataract, eye injury, glaucoma, diabetic retinopathy, or macular edema, (3) coexisting or history of severe systemic diseases other than well-controlled diabetes and hypertension, (4) history of pars plana vitrectomy, posterior scleral reinforcement, and laser photocoagulation treatment, (5) evidence of retinal pathology non-related to myopia, and (6) poor-quality images for MM or counting PSV grading.

### Definition and Classification of MM

Based on the new classification and grading system for MM (ATN), MM was subdivided into 3 types: myopic atrophy maculopathy (MAM), myopic tractional maculopathy (MTM), and myopic neovascular maculopathy (MNM) (11). A scores were based on 5 levels: A0 (no myopic retinal lesions, A1 (tessellated fundus only), A2 (diffuse chorioretinal atrophy), A3 (patchy chorioretinal atrophy), and A4 (complete macular atrophy). MAM was defined as an A score of ≥2. T score had 6 levels: T0 (no macular schisis), T1 (inner or outer foveoschisis) T2, inner and outer foveoschisis, T3 (foveal retinal detachment), T4 (full-thickness macular hole [MH]), and T5 (MH and retinal detachment), and MTM were defined as T score ≥1. N score was divided into 4 levels (N0, no mCNV, N1 (macular LCs), N2a (active CNV), and N2s (scar or Fuch's spot), and MNM defined as an N score ≥1. The classification and grading of MM were done independently by two well-trained graders (JFS and HL). Disagreements were adjudicated by a retinal specialist (XFS).

### Spectral-Domain OCT Imaging and Assessment

**Figure 1:**
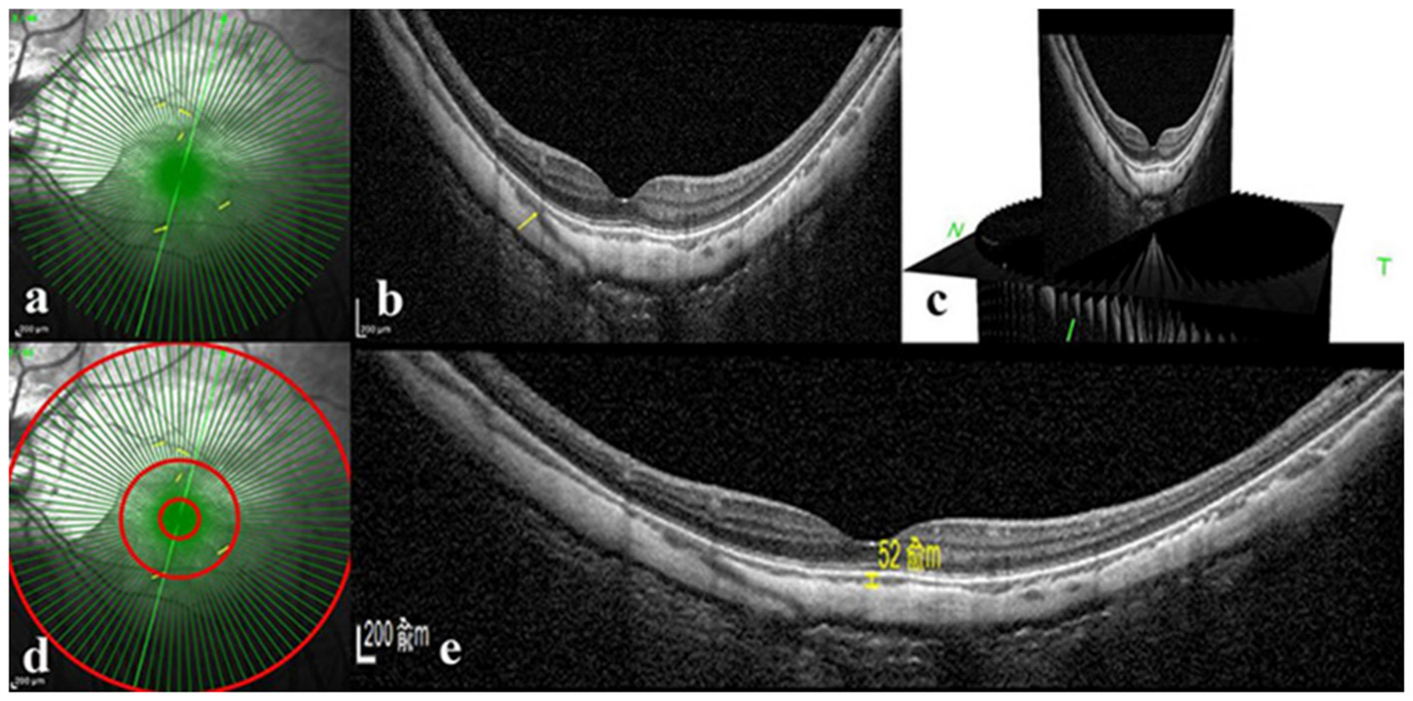
Perforating scleral vessel counting method and macular choroidal thickness assessment using spectral-domain OCT. **(a)** Structural OCT en face image of one patient. Enhanced depth OCT scanning was centered on the macular with 48 radical scans, a length of 9 mm and an A-scan repeat time of 9. **(b)** B-scan image corresponding to the scan line of the green arrow in part a. The yellow arrow in parts a and b indicates the corresponding position where PSV entered the choroid from the sclera. **(c)** Three-dimensional reconstruction picture of B-scan image based on Heidelberg software. **(d)** Counting of PSV in three different regions, such as 1 diameter circle, 3 diameter circle, and 9 diameter circle. **(e)** Structural OCT scan through the fovea. Macular choroidal thickness is measured in a 1:1 μm image using Heidelberg software (shown as a yellow line segment of 52 μm). OCT: optical coherence tomography.

Macular-centered structural OCT images were obtained for each eye using enhanced depth imaging with 48 radial scans, a length of 9 mm, and an A-scan repeat time (ART) of 9. Macular choroidal thickness (mChT) was defined as the distance between the Bruch membrane and the choroid-sclera interface in the fovea. PSV was determined by 2 experienced retinal physicians (HMY and JFS) using the marking software (Heidelberg Engineering, Heidelberg, Germany). Based on the study by Giuffrè et al. (8), PSV was defined as (1) linear or wavy morphology in OCT images, (2) hyporeflective appearance, and (3) extension from the sclera through the choroid toward the retina. In OCT B-scan, the PSV mark was taken at the junction of the sclera and choroid, and at the corresponding site in the OCT en face image (Figure 1). The continuous 48 equally spaced radial OCT B-scans and three-dimensional picture were used to avoid duplicated counting by demonstrating how PSV passed through the sclera and entered the choroid (Figure 1). Since our study aimed to explore the maculopathy, PSVs within central 1, 3, and 9 mm diameter circles were counted.

### Statistical Analysis

We first compared data distribution for each covariate between bilateral eyes, MM, and non-MM groups, among the MAM, MTM, and MNM groups. *T*-test (normal distribution) or Kruskal-Wallis rank-sum test (non-normal distribution) were used for continuous variables. Chi-square tests were used for categorical data (Tables 1–3). Univariate and multivariate logistic regression models were used to determine if PSV count correlated with MM (Table 4; Supplementary Table S1). Total PSV count was obtained in 3 different regions, separately. Stratified analysis was used to verify the relationships in different sub-groups (Table 5). All analyses were done on R (http://www.R-project.org) and EmpowerStats software (www.empowerstats.com, X&Y solutions, Inc. Boston, MA, USA).

**Table 1:** General characteristics of participants with and without myopic maculopathy.

**Variables**	**Total cohort**	**Non-MM group**	**MM group**	***P*-value**
No. of eyes (%)	188	59	129	
Age, years	43.36 ± 15.27	32.34 ± 12.42	48.40 ± 13.75	<0.001
AL, mm	29.10 ± 2.05	27.75 ± 1.06	29.72 ± 2.10	<0.001
mChT, μm	74.61 ± 52.80	122.52 ± 45.08	52.90 ± 40.31	<0.001
Total PSV count	7.59 ± 2.95	9.47 ± 2.68	6.73 ± 2.66	<0.001
Gender, no (%)				0.234
Female	123 (65.43%)	35 (59.32%)	88 (68.22%)	
Male	65 (34.57%)	24 (40.68%)	41 (31.78%)	
Cilioretinal artery, no (%)				0.234
No	175 (93.09%)	53 (89.83%)	122 (94.57%)	
Yes	13 (6.91%)	6 (10.17%)	7 (5.43%)	
HBP, no (%)				0.015
No	176 (93.62%)	59 (100.00%)	117 (90.70%)	
Yes	12 (6.38%)	0 (0.00%)	12 (9.30%)	
DM, no (%)				0.311
No	184 (97.87%)	59 (100.00%)	125 (96.90%)	
Yes	4 (2.13%)	0 (0.00%)	4 (3.10%)	
Anti-VEGF, no (%)				0.006
No	173 (92.02%)	59 (100.00%)	114 (88.37%)	
Yes	15 (7.98%)	0 (0.00%)	15 (11.63%)	

*MM, myopic maculopathy; AL, axial length; mChT, macular choroidal thickness; PSV, perforating scleral vessel; HBP, high blood pressure; DM, diabetes mellitus; VEGF, vascular endothelial growth factor. p-values for the difference in variables between the non-MM and MM groups are based on Wilcoxon rank-sum test or chi-square test, as appropriate*.

**Table 2:** Characteristics and comparison between highly myopic eyes and three different types of myopic maculopathy.

**Variables**	**MAM group**	**MTM group**	**MNM group**	***P*-value**
No. of eyes	97	64	90	
Age, years	48.96 ± 14.01	54.12 ± 12.89	49.51 ± 13.75	0.072
AL, mm	30.12 ± 1.98	30.20 ± 2.06	30.17 ± 2.07	0.977
mChT, μm	44.34 ± 34.21	40.13 ± 30.57	46.34 ± 37.22	0.578
Total PSV count	6.29 ± 2.72	5.92 ± 2.28	6.09 ± 2.47	0.794
Gender, no (%)				0.095
Female	68 (70.10%)	53 (82.81%)	61 (67.78%)	
Male	29 (29.90%)	11 (17.19%)	29 (32.22%)	
Cilioretinal artery				0.974
No	92 (94.85%)	61 (95.31%)	86 (95.56%)	
Yes	5 (5.15%)	3 (4.69%)	4 (4.44%)	
HBP				0.653
No	86 (88.66%)	54 (84.38%)	80 (88.89%)	
Yes	11 (11.34%)	10 (15.62%)	10 (11.11%)	
DM				0.985
No	86 (88.66%)	54 (84.38%)	80 (88.89%)	
Yes	11 (11.34%)	10 (15.62%)	10 (11.11%)	
Anti-VEGF				0.294
No	87 (89.69%)	58 (90.62%)	75 (83.33%)	
Yes	10 (10.31%)	6 (9.38%)	15 (16.67%)	
MAM	/	51 (79.69%)	68 (75.56%)	
MTM	51 (52.58%)	/	46 (51.11%)	
MNM	68 (70.10%)	46 (71.88%)	/	

*MAM, myopic atrophic maculopathy; MTM, myopic tractional maculopathy; MNM, myopic neovascular maculopathy; VEGF, vascular endothelial growth factor. p-values for the difference in variables among MAM, MTM, and MNM groups based on Wilcoxon rank-sum test or chi-square test, as appropriate*.

**Table 3:** PSV count in different regions and comparison among different myopic groups.

**PSV count**	**Total cohort**	**Non-MM**	**MM**	**MAM**	**MTM**	**MNM**	**Pa**	**Pb**
Total	7.59 ± 2.95	9.47 ± 2.68	6.73 ± 2.66	6.29 ± 2.72	5.92 ± 2.28	6.09 ± 2.47	<0.001	0.794
Fovea	0.29 ± 0.51	0.29 ± 0.49	0.29 ± 0.52	0.29 ± 0.54	0.23 ± 0.50	0.22 ± 0.44	0.871	0.726
Parafovea	1.80 ± 1.18	1.93 ± 1.27	1.74 ± 1.14	1.68 ± 1.14	1.64 ± 1.12	1.68 ± 1.12	0.425	0.977
Perifovea	5.51 ± 2.68	7.25 ± 2.60	4.71 ± 2.3	4.32 ± 2.29	4.05 ± 2.00	4.19 ± 2.23	<0.001	0.742

*Pa value for the difference in variables between the non-MM group and MM group. Pb value for the difference in variables among MAM, MTM, and MNM groups. MAM, myopic atrophic maculopathy; MTM, myopic tractional maculopathy; MNM, myopic neovascular maculopathy*.

**Table 4:** Relationship between PSV count and three different types of myopic maculopathy.

**PSV count**	**Non-adjusted**		**Adjust I**		**Adjust II**	
	**OR (95%CI)**	***P***	**OR (95%CI)**	***P***	**OR (95%CI)**	***P***
**MAM**
Total	0.67 (0.58, 0.77)	<0.0001	0.90 (0.76, 1.07)	0.2452	0.90 (0.75, 1.07)	0.2419
Fovea	1.01 (0.58, 1.78)	0.9683	1.81 (0.84, 3.91)	0.1318	1.87 (0.85, 4.07)	0.1175
Parafovea	0.84 (0.66, 1.07)	0.1610	0.99 (0.70, 1.38)	0.9333	1.00 (0.71, 1.41)	0.9871
Perifovea	0.64 (0.55, 0.75)	<0.0001	0.86 (0.71, 1.04)	0.1158	0.85 (0.70, 1.03)	0.1028
**MTM**
Total	0.69 (0.60, 0.80)	<0.0001	0.93 (0.77, 1.12)	0.4421	0.94 (0.77, 1.15)	0.5678
Fovea	0.72 (0.39, 1.35)	0.3075	0.95 (0.42, 2.14)	0.8993	1.06 (0.47, 2.40)	0.8935
Parafovea	0.84 (0.65, 1.09)	0.1915	0.92 (0.64, 1.33)	0.6718	0.94 (0.64, 1.38)	0.7609
Perifovea	0.68 (0.59, 0.79)	<0.0001	0.94 (0.77, 1.16)	0.5604	0.95 (0.76, 1.18)	0.6267
**MNM**
Total	0.64 (0.55, 0.74)	<0.0001	0.81 (0.68, 0.96)	0.0180	0.78 (0.64, 0.95)	0.0149
Fovea	0.61 (0.34, 1.09)	0.0962	0.76 (0.37, 1.58)	0.4687	0.81 (0.37, 1.77)	0.5992
Parafovea	0.85 (0.66, 1.08)	0.1833	0.94 (0.68, 1.29)	0.7000	0.90 (0.63, 1.27)	0.5458
Perifovea	0.63 (0.53, 0.73)	<0.0001	0.81 (0.67, 0.97)	0.0256	0.80 (0.65, 0.98)	0.0299

*ORs were derived from multivariate logistic regression analysis. Adjust I model adjusted for age, AL, mChT, gender, and cilioretinal artery. Adjust II model adjusted for age, AL, mChT, gender, cilioretinal artery, HBP, DM, and anti-VEGF treatment. mChT, macular choroidal thickness; AL, axial length; HBP, high blood pressure, DM, diabetes mellitus; VEGF, vascular endothelial growth factor*.

**Table 5:** Stratified analysis of the association between perforating scleral vessel and myopic neovascular maculopathy.

**Sub-group**	***N***	**Odds ratio (95% CI)**	***P*-value**
X = No. PSV			
Gender
Female	123	0.66 (0.56, 0.79)	<0.0001
Male	65	0.58 (0.44, 0.77)	0.0002
Age, years
<35	64	0.63 (0.46, 0.87)	0.0045
≥35, <55	79	0.72 (0.59, 0.88)	0.0016
≥55	45	0.67 (0.46, 0.97)	0.0392
AL, mm
<28	70	0.88 (0.69, 1.11)	0.3497
≥28, <30	55	0.41 (0.25, 0.65)	0.0002
≥30	63	0.75 (0.58, 0.96)	0.0254
mChT, μm
<50	79	0.76 (0.59, 1.00)	0.0680
≥50, <100	49	0.65 (0.48, 0.87)	0.0045
≥100	58	1.01 (0.71, 1.43)	0.9754
Cilioretinal artery
No	175	0.64 (0.55, 0.75)	<0.0001
Yes	13	0.55 (0.23, 1.31)	0.1758

*Odds ratios were derived from multivariate logistic regression analysis and adjusted for gender, age, AL, mChT, cilioretinal artery, HBP, DM, and anti-VEGF. mChT, macular choroidal thickness; AL, axial length; HBP, high blood pressure; DM, diabetes mellitus; VEGF, vascular endothelial growth factor*.

## Results

No significant differences in bilateral ocular biometrics, such as AL, mChT, and anti-vascular endothelial growth factor (VEGF) history, were revealed by the generalized equation regression models. Therefore, it was unnecessary to adjust for associations between the two eyes. General characteristics among participants with and without MM are shown in Table 1. The dataset was comprised of 188 eyes (117 participants). Mean age, mean AL, mean mChT, and mean PSV number were 43.36 ± 15.27 years, 29.10 ± 2.05 mm, 74.61 ± 52.80 μm, and 7.59 ± 2.95, respectively. Of the eligible eyes, 65 (34.57%) were male, 13 (6.91%) had cilioretinal artery, and 15 (7.98%) had a history of intravitreal anti-VEGF injection. In comparison with the non-MM group, MM group was characterized by older age (48.4 vs. 32.34, *p* < 0.001), longer AL (29.72 vs. 27.75, *p* < 0.001), thinner mChT (52.90 vs. 122.52, *p* < 0.001), and lower PSV count (6.73 vs. 9.47, *p* < 0.001). Other variables, such as gender, cilioretinal artery, and diabetes mellitus (DM), did not differ significantly between the non-MM group and MM group.

**Figure 2:**
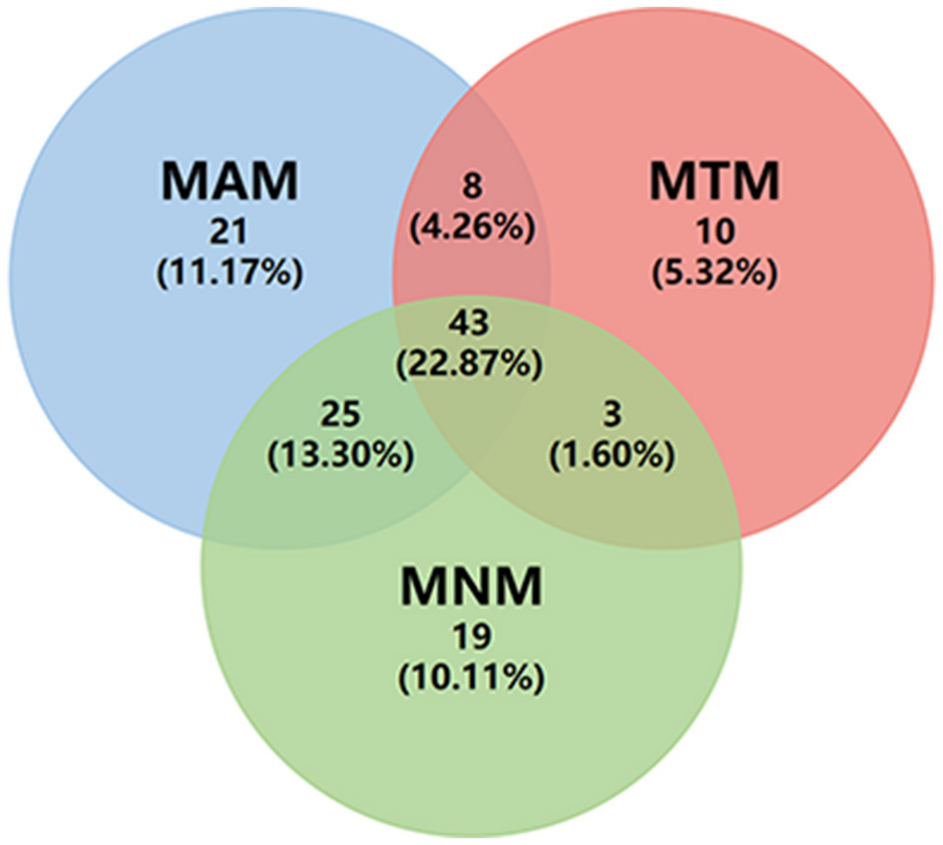
Venn diagram of eye count in three different types of myopic maculopathy. MAM, myopic atrophic maculopathy; MTM, myopic tractional maculopathy; MNM, myopic neovascular maculopathy.

**Figure 3:**
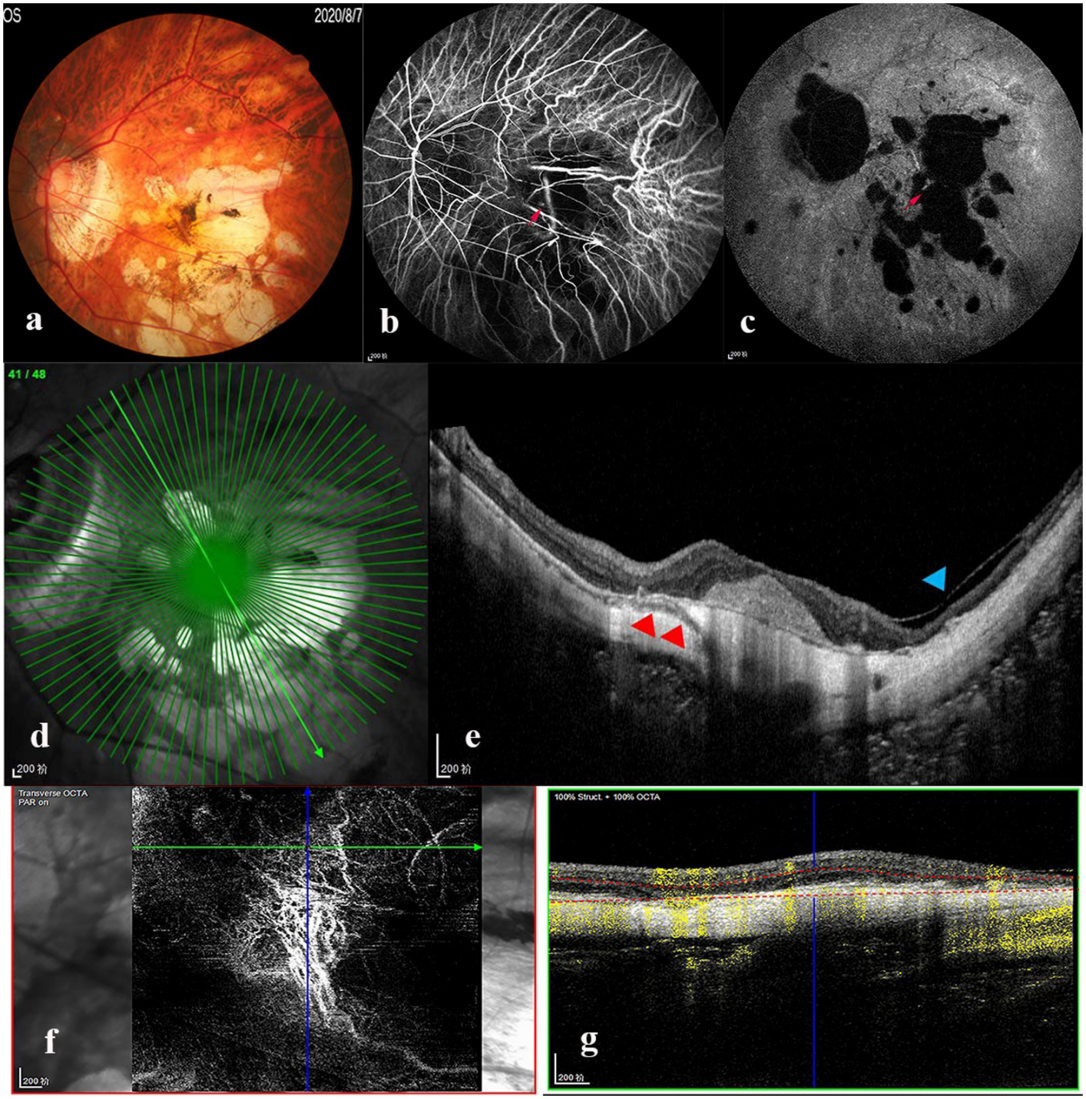
Multimode imaging of a patient who combined the three types of myopic maculopathy. Left eye images of a patient aged 70–75 years old, with an axial length of 29–30 mm. **(a)** Color fundus photograph showed macular atrophy (MAM) and Fuch's spot (MNM). Fluorescence leakage (red arrow) was seen in the early **(b)** and late **(c)** stages of fluorescence angiography. **(d,e)** OCT image demonstrated foveoschisis (MTM, blue arrowhead) and PSV (red arrowhead) beneath the choroidal neovascularization (CNV). **(f,g)** OCT-angiography showed CNV (MNM) formation. MAM, myopic atrophic maculopathy; MTM, myopic tractional maculopathy; MNM, myopic neovascular maculopathy; OCT, optical coherence tomography.

Myopic maculopathy was divided into MAM, MTM, and MNM (Table 2). The 3 groups did not differ significantly with regards to age, AL, mChT, total PSV count, gender, cilioretinal artery, high blood pressure (HBP), DM, and anti-VEGF treatment history. Venn diagram analysis was used to show the number and proportion of eyes in the 3 groups (Figure 2). Among them, the proportion of eyes that combined MAM, MTM, and MNM was the largest (22.87%), and the proportion of eyes that combined MTM and MNM was the least (1.60%). Figure 3 shows a representative left eye that combined 3 kinds of MMs, such as macular atrophy, foveoschisis, CNV, and Fuch's spot, from a patient aged 70–75 years old, with an AL of 29–30 mm. Table 3 describes PSV count in separate regions. In the total cohort, PSV count was 7.59 ± 2.95 in 0–9 mm total area, 0.29 ± 0.51 in 0–1 mm fovea area, 1.80 ± 1.18 in 1–3 mm parafovea area, and 5.51 ± 2.68 in 3–9 mm perifovea area. Among MAM, MTM, and MNM groups, PSV count in different regions had no significant difference. However, the non-MM group had higher PSV counts in total area (0–9 mm, 9.47 vs. 6.73, *p* < 0.001) and perifovea area (3–9 mm, 7.25 vs. 4.71, *p* < 0.001) relative to the MM group. Univariate and multivariate regression analyses revealed that there were no significant associations between PSV number in different regions with MAM (total *p* = 0.2419, fovea *p* = 0.1175, parafovea *p* = 0.9871, and perifovea *p* = 0.1028) and MTM (total *p* = 0.5678, fovea *p* = 0.8935, parafovea *p* = 0.7609, and perifovea *p* = 0.6267). Total PSV count [odds ratio (OR) 0.78, 95% CI 0.64–0.95, *p* = 0.0149] and perifovea PSV count (OR 0.80, 95% CI 0.65–0.98, *p* = 0.0299) were both protective factors for MNM. AL (OR 1.32, 95% CI 1.02–1.71, *p* = 0.0322) and mChT (OR 0.98, 95% CI 0.97–0.99, *p* = 0.0014) strongly correlated with MAM (Supplementary Table S1).

The stratified analysis demonstrated that regardless of subgroup, PSV count was negatively associated with MNM, with ORs ranging from 0.41 to 0.75 (Table 5). However, in patients with AL <28 mm, mChT <50 μm, mChT ≥100 μm, and eyes with cilioretinal artery, efficacies of PSV count were greatly attenuated (OR = 0.69–1.11, 0.59–1.00, 0.71–1.43, and 0.23–1.31, respectively).

## Discussion

To our knowledge, this is the largest study demonstrating an association between PSV and 3 kinds of MMs. The highlight of this study was the quantitative analysis of PSV based on a large number of Chinese patients. We find that in groups with AL ≥28 mm or mChT 50–100 μm, or without cilioretinal artery, the greater number of PSV, the lower probability of MNM. If confirmed, these findings would help to identify individuals who are at high risk of developing MNM and determine the role of sclera and choroid in the pathophysiological mechanisms of myopia from the vascular perspective.

Differences between the non-MM and MM groups were consistent with previous studies. The prevalence of myopia was increased with age, and the progression of macular lesions in highly myopic eyes was correlated with age (2, 12). Other studies also reported higher AL and thinner mChT in PM groups compared to the simple myopia group (13, 14).

In our study, the stratified analysis revealed that clinical relevance between PSV and MNM was significant only in patients without cilioretinal artery, suggesting that cilioretinal arteries may protect from myopic progression, which is consistent with past findings. Zhu et al. (15) observed that adults with cilioretinal arteries had higher macular vessel density compared to those without. Meng et al. (16) found the presence of cilioretinal arteries may correlate with better visual function in myopic eyes.

Past studies reported that PSV may be an anatomical weakness in myopic eyes causing PM complications. PSV influences the formation of Lc and CNV (5–7, 9). In eyes with myopic patchy atrophy, PSV contributes to myopic morphological changes, such as sclera bowing and atrophy progression (10). However, in previous studies, only the probability of PSV occurrence under specific myopic complications was calculated, and factors, such as AL, mChT, gender, cilioretinal artery, illness, and treatment history, were not controlled for in statistical analyses. Hence, we performed a deeper analysis based on large sample size, and our conclusion was valid in certain groups based on stratified analysis.

The study by Giuffrè et al. (8) included PSV number per eye and PSV number per CNV as indicators of the relationship between PSV and CNV and found that the average PSV number per eye was 2.1 ± 1.0 and that PSV often coincided with CNV. However, the sample size of the study was quite small (41 eyes from 39 patients), and the scanning area was not specifically defined. The study by Rothenbuehler et al. (17) counted PSV in 22 emmetropic eyes based on 3D imaging and found the PSV for the central 1 mm diameter and 3 mm diameter to be 0.2 ± 0.5 (range 0–2) and 2.1 ± 1.8 (range 0–7), respectively, which is consistent with our data on highly myopic eyes without MM (0.29 ± 0.49 for central 1 mm diameter, 1.93 ± 1.27 central 3 mm diameter). In contrast, our study described PSV distribution better, using a larger scanning scope (9 mm diameter), and revealed the predictive effect of PSV on MM based on ATN staging.

For the association between PSV and MAM, Xie et al. showed that PSV was associated with sclera bowing and atrophy progression in eyes with patchy atrophy (10). Patchy atrophy, which rarely involved the fovea but always preferentially occurred inferior and temporal to the fovea, is one type of MAM lesion (A3 degree) (10, 18). Comparably, our study focused on the macular circle region for a large area (central 9 mm diameter) and all types of MAM lesions (not only patchy atrophy but also tessellated, diffuse, macular atrophy) were included. Additionally, our multivariate analysis showed that AL (OR 1.32, 95% CI 1.02–1.71, *p* = 0.0322) and mChT (OR 0.98, 95% CI 0.97–0.99, *p* = 0.0014) strongly correlated with MAM and attenuated the efficacy of PSV count (Supplementary Table S1).

The protective effect of PSV on MNM could be explained by vascular ischemic theory. AL elongation resulting in stretching and thinning of the choroid and sclera is a commonly accepted mechanism of PM. In high myopic eyes, the area of flow deficit in the choriocapillaris layer was significantly greater than that in emmetropes and mild or moderate myopic eyes (19, 20). Animal models revealed that choroidal thickness and choroidal blood perfusion are decreased in myopic eyes, and hypoxia-signaling is activated in the myopic sclera and these events can be targeted for myopia control (21, 22). PSV was confirmed to be SPCA, which mainly supplies choroid blood flow. Additionally, PSV may be crucial for oxygen diffusion to the sclera. Thus, metabolic insufficiency due to less PSV may cause hypoxia to the choroid and sclera, and trigger MNM.

The strength of our study is in its large sample size and rigorous statistical analyses. However, it has some limitations. First, due to poor penetration using the SD-OCT technique, it was not feasible to count PSVs in eyes with mChT ≥200 μm. Thus, our study population was mainly comprised of patients with thin choroids. Secondly, PSV counting was manual and may be inaccurate even counting was independently done by 2 researchers. Thirdly, we have not experimentally verified this finding, for instance, using animal models to verify differences between signaling pathways in PSV angiogenesis.

## Data Availability Statement

The datasets presented in this study can be found in online repositories. The names of the repository/repositories and accession number(s) can be found below: http://www.medresman.org.cn.

## Ethics Statement

The studies involving human participants were reviewed and approved by the Medical Ethics Committee of Tongji Hospital, Tongji Medical College, Huazhong University of Science and Technology. The patients/participants provided their written informed consent to participate in this study.

## Author Contributions

XS and HY: conception or design of the work. HY, JS, HL, and ZW: data collection. HY and JS: data analysis and interpretation. HY: drafting the article. XS, JS, and HL: critical revision of the article. HY, JS, HL, ZW, and XS: final approval of the version. All authors contributed to the article and approved the submitted version.

## Funding

This work was supported by grants from the National Natural Science Foundation of P.R. China (Grant No. 81974136) and the Health Commission of Hubei Province Scientific Research Project (Grant No. WJ2019M139).

## Conflict of Interest

The authors declare that the research was conducted in the absence of any commercial or financial relationships that could be construed as a potential conflict of interest.

## Publisher's Note

All claims expressed in this article are solely those of the authors and do not necessarily represent those of their affiliated organizations, or those of the publisher, the editors and the reviewers. Any product that may be evaluated in this article, or claim that may be made by its manufacturer, is not guaranteed or endorsed by the publisher.
