# Supplementary material for: Association Between Perforating Scleral Vessel and Myopic Maculopathy: A Cross-Sectional Study of a Chinese Cohort
Source: Front Med (Lausanne). 2022 Jan 6;8:727680. doi: 10.3389/fmed.2021.727680 (PMC8770412; doi:10.3389/fmed.2021.727680)
Supplement: Supplementary file 1 [file Table_1.docx]

**Supplementary Table S1**

**Multivariate analysis of risk factors on myopic atrophic maculopathy**

| Exposure | Odds ratio (95%CI) | P value |
| --- | --- | --- |
| Gender |  |  |
| Female | 1.0 |  |
| Male | 0.95 (0.41, 2.23) | 0.9149 |
| Age, years | 1.01 (0.98, 1.05) | 0.3701 |
| AL, mm | 1.32 (1.02, 1.71) | 0.0322 |
| mChT, μm | 0.98 (0.97, 0.99) | 0.0014 |
| No. of PSV | 0.90 (0.75, 1.07) | 0.2419 |
| HBP |  |  |
| No | 1.0 |  |
| Yes | 5.07 (0.53, 48.60) | 0.1588 |
